# The Comparison of the Dietary Intake Loss Between Elderly and Non-Elderly Patients After Gastrectomy for Gastric Cancer

**DOI:** 10.1007/s12029-021-00776-x

**Published:** 2022-01-03

**Authors:** Masato Nakazono, Toru Aoyama, Keisuke Komori, Hayato Watanabe, Kazuki Kano, Shinsuke Nagasawa, Kenki Segami, Hiroshi Tamagawa, Norio Yukawa, Yasushi Rino, Takashi Ogata, Takashi Oshima

**Affiliations:** 1 Department of Gastrointestinal Surgery, Kanagawa Cancer Center, 2-3-2 Nakao, Asahi-ku, Yokohama, 241-8515 Japan; 2 Department of Surgery, Yokohama City University, Yokohama, Japan

**Keywords:** Gastric cancer, Gastrectomy, Elderly, Dietary intake, Nutrition

## Abstract

**Background/Aim:**

The present study quantified the changes in the dietary and nutritional intake after gastrectomy between elderly and non-elderly patients.

**Patients and Methods:**

This prospective observational study enrolled patients who underwent curative gastrectomy for gastric cancer.

**Results:**

Twenty-three patients ≥ 75 years old were classified into the elderly group, and 127 patients < 75 years old were classified into the non-elderly group. The respective median % dietary intake losses at 1 and 3 months postoperatively were −12.4% and −5.3% in the elderly group and −8.3% and −2.8% in the non-elderly group (*p* = 0.075 and 0.080). On comparing the intake loss of three major nutrients, the respective median % lipid intake losses at 1 and 3 months postoperatively were −13.5% and −5.8% in the elderly group and −7.3% and 0% in the non-elderly group (*p* = 0.029 and 0.045).

**Conclusion:**

Our results suggested that elderly patients experienced more serious lipid intake loss after gastrectomy than non-elderly patients.

## Background

Gastrectomy is an essential treatment for gastric cancer [1–3]. In recent years, the number of elderly patients has been rapidly increasing worldwide [4]. With this social background, opportunities for elderly patients to undergo gastrectomy for gastric cancer have increased.

Previous studies showed that elderly patients tended to experience a greater loss of lean body mass and body weight than non-elderly patients [5]. This body composition change is a common and important problem after gastrectomy for gastric cancer [6, 7], as changes in the body composition after gastrectomy reduce the nutritional status, postoperative quality of life, and compliance with adjuvant chemotherapy, all of which can lead to a poor survival [8–10].

Various factors potentially underlying the changes in body composition after gastrectomy have been considered, including a decreased dietary intake (DI) due to the loss of the reservoir function, malabsorption, hyper-catabolism due to surgical stress, lack of exercise, and a reduction in the blood ghrelin level [11–15]. Among them, a decreased DI has been considered a predominant factor in the body composition changes after gastrectomy [14, 15]. Therefore, we hypothesized that elderly patients experienced much greater DI loss after gastrectomy than non-elderly patients.

However, the changes in DI after gastrectomy in elderly patients have not been objectively reported. How much DI loss is experienced by elderly patients after gastrectomy compared with non-elderly patients and whether or not the nutritional intake differs markedly between elderly and non-elderly patients has been unclear.

The primary aim of the present study was to quantify the changes in the DI in elderly patients after gastrectomy compared with non-elderly patients using the Food Frequency Questionnaire with 82 food items (FFQW82). The secondary aim was to compare the changes in the intake of three major nutrients (sugar, protein, and lipid) after gastrectomy between elderly and non-elderly patients.

## Materials and Methods

### Patient

This prospective observational study was conducted from May 2011 to November 2014 at Kanagawa Cancer Center. The eligibility criteria were as follows: (1) histologically proven gastric adenocarcinoma diagnosed as pathological stage IA or IB according to the Japanese Classification of Gastric Carcinoma [16]; and (2) received nutritional counseling 2 to 4 days before surgery and 1 and 3 months postoperatively. Patients who received preoperative or postoperative chemotherapy were excluded. Because the patients who received the perioperative chemotherapy suffered the adverse event, such as anorexia, nausea, and vomiting. Anorexia, nausea, and vomiting affect for the patient’s oral intake. Thus, we excluded the patients who received the perioperative chemotherapy in the present study. The patients were classified into groups < 75 years old (non-elderly group) and ≥ 75 years old (elderly group).

### Perioperative Care

All patients received perioperative care using the enhanced recovery after surgery (ERAS) protocol. The details of this protocol have been reported in a previous study [17, 18]. In brief, patients were able to eat a normal diet until dinner the day before surgery and drink a rehydration solution until 3 h before surgery. Premedication was not administered. The nasogastric tube was removed immediately after surgery. Oral intake was initiated on postoperative day (POD) 2, beginning with water and an oral nutritional supplement. The patients began soft diet intake on POD 3 and progressed to eating regular food every 2 days (3 steps). The patients were discharged when they had successfully achieved adequate pain relief and soft food intake, had returned to their preoperative mobility level, and exhibited normal laboratory data on POD7. In the present study, we did not use the nutritional supplement.

### Study Schedule

The patients received perioperative nutritional counseling on the day of hospitalization, the day of discharge, and 1 and 3 months postoperatively. In counseling during hospitalization, which was 2 to 4 days before surgery, nutritionists analyzed patients’ preoperative DI conditions and measured their body weight. In counseling at discharge, nutritionists provided education on how to eat meals after gastrectomy, including (but not limited to) increasing the intake frequency, decreasing the portion size at meals, chewing frequently, and eating slowly. In counseling at 1 and 3 months postoperatively, nutritionists analyzed the patients’ postoperative DI conditions, measured their body weight, and provided education on how the patients could improve their nutritional status based on the results.

### The Analysis of the Dietary Intake

The DI was evaluated at perioperative nutritional counseling using the FFQW82. The FFQW82 was established and validated by Watanabe and Adachi et al. in 2011 [19]. The FFQW82 is a self-administered questionnaire that is designed to present 82 food items according to 16 food groups and inquire about the intake frequency and portion size of each meal. The intake frequency is indicated by six categories: (0 = “absolutely do not eat”; 1 = “eat once or twice per month”; 2 = “eat once or twice per week”; 3 = “eat 3 to 4 times per week”; 4 = “eat 5 to 6 times per week”; 5 = “eat everyday”). The portion size is described as “small,” “medium,” or “large.” The standard amount for “medium” is shown by size on the food list with pictures. “Small” is defined as half the amount of “medium,” whereas “large” is defined as 1.5 times the amount of “medium.” Patients completed the FFQW82 in approximately 30 min. Based on the responses to the FFQW82, the estimated intake of energy and several nutrients for a whole day were calculated by simply summing up the estimated intake of food items for each meal. Microsoft® Excel (Microsoft Inc., Redmond, WA, USA) was used to obtain the nutrient composition for items on the FFQW82.

### Evaluations, Statistical Analyses, and Ethics

DI loss was defined as % DI loss = (DI at 1 month postoperatively and 3 months postoperatively—preoperative DI) × 100/preoperative DI. Intake loss of three major nutrients (NI loss) was defined as % of each NI loss = (each NI at 1 month postoperatively and 3 months postoperatively—preoperative each NI) × 100/preoperative each NI. The values were expressed as the median and range.

The data were compared between the non-elderly and elderly groups using the chi-squared test and the Mann–Whitney *U* test. *P* values of < 0.05 were considered to indicate statistical significance. Analyses were performed using the SPSS version 25.0 software program (Statistical Package for the Social Science; SPSS, Chicago, IL, USA).

This study was approved by the Institutional Review Board of Kanagawa Cancer Center (2020 epidemiologic study-163). The study was conducted in accordance with the Declaration of Helsinki.

## Results

### Patient Characteristics

A total of 150 patients were examined in this study. The median age (range) of the 150 patients was 67 (27–86) years old. A total of 95 patients were male, and 55 were female. The 127 patients < 75 years old were classified into the non-elderly group, and the 23 patients ≥ 75 years old were classified into the elderly group.

The patient characteristics are shown in Table 1. The preoperative height and body weight were similar for the two groups. The American Society of Anesthesiology score tended to be worse and the incidence of hypertension significantly higher in the elderly group than in the non-elderly group (*p* = 0.07, and *p* = 0.009, respectively). The incidence of diabetes mellitus and chronic obstructive pulmonary disease were similar between the two groups.

**Table 1:** Comparison of the patients’ background

	All cases	Non-elderly group (< 75 years)	Elderly group (≥ 75 years)	*P* value
	Number of patients (%)	Number of patients (%)	Number of patients (%)	
	*N* = 150	*N* = 127	*N* = 23	
Age, years*	67 (27–86)	65 (27–74)	78 (75–86)	< 0.001
Gender				0.50
Male	95 (63.3)	79 (62.2)	16 (69.6)	
Female	55 (36.7)	48 (37.8)	7 (30.4)	
Height, cm*	162.2 (132.8–182.8)	162.1 (132.8–182.8)	162.2 (142.3–175.0)	0.232
Body weight, kg*	59.1 (35.3–88.2)	58.8 (35.3–88.2)	59.1 (36.9–73.8)	0.884
Body mass index*	22.3 (15.6–31.8)	22.2 (15.6–31.8)	22.5 (16.7–27.3)	0.733
ASA-PS				0.07
1	48 (32.0)	47 (37.0)	1 (4.3)	
2	99 (66.0)	78 (61.4)	21 (91.4)	
3	3 (2.0)	2 (1.6)	1 (4.3)	
Co-morbidity				
Hypertension	61 (40.7)	46 (36.2)	15 (65.2)	0.009
Diabetes mellitus	16 (10.7)	12 (9.4)	4 (17.4)	0.272
COPD	11 (7.3)	10 (7.9)	1 (4.3)	1.00
Clinical stage				1.00
IA-IB	140 (93.3)	118 (92.9)	22 (95.7)	
IIA-IIB	10 (6.7)	9 (7.1)	1 (4.3)	
Clinical T factor				0.667
T1a-2	139 (92.7)	118 (92.9)	21 (91.3)	
T3-4a	11 (7.3)	9 (7.1)	2 (8.7)	
Clinical N factor				1.00
N0	148 (98.7)	125 (98.4)	23 (100)	
N1	2 (1.3)	2 (1.6)	0 (0)	

*ASA-PS* American Society of Anesthesiologists physical status, *COPD* chronic obstructive pulmonary disease

^*^Median (range)

### Surgical and Pathological Outcomes

The procedure, lymph node dissection, type of approach, type of reconstruction, operation time, and bleeding amount were similar for the two groups. The complications were classified according to the Clavien-Dindo classification [20], and the rate of complications over grade 2 was 13.4% in the non-elderly group and 17.4% in the elderly group. There were no significant differences between the groups (*p* = 0.533). The pathological outcomes of the two groups did not differ to a statistically significant extent (Table 2).

**Table 2:** The surgical and pathological outcomes

	All cases	Non-elderly group (< 75 years)	Elderly group (≥ 75 years)	*P* value
	Number of patients (%)	Number of patients (%)	Number of patients (%)	
	*N* = 150	*N* = 127	*N* = 23	
Procedure				0.974
Distal gastrectomy	117 (78.0)	99 (78.0)	18 (78.2)	
Total gastrectomy	33 (22.0)	28 (22.0)	5 (21.8)	
Lymph node dissection				1.00
D1 + dissection	126 (84.0)	106 (83.5)	20 (87.0)	
D2 dissection	24 (16.0)	21 (16.5)	3 (13.0)	
Type of approach				0.931
Conventional	51 (34.0)	43 (33.9)	8 (34.8)	
Laparoscopic	99 (66.0)	84 (66.1)	15 (65.2)	
Type of reconstruction				0.846
Billroth-I	94 (62.7)	80 (63.0)	14 (60.9)	
Roux-en-Y	56 (37.3)	47 (37.0)	9 (39.1)	
Operation time, min*	270 (85–512)	270 (85–512)	267 (113–461)	0.633
Bleeding, g*	67.5 (0–950)	70 (0–950)	65 (5–530)	0.733
Postoperative complications**	21 (14.0)	17 (13.4)	4 (17.4)	0.533
Pathological stage				0.223
IA	125 (83.3)	108 (85.0)	17 (73.9)	
IB	25 (16.7)	19 (15.0)	6 (26.1)	
Pathological T factor				0.319
1	136 (90.7)	117 (92.1)	19 (82.6)	
2	14 (9.3)	10 (7.9)	4 (17.4)	
Pathological N factor				0.677
0	139 (92.7)	118 (92.9)	21 (91.3)	
1	11 (7.3)	9 (7.1)	2 (8.7)	

^*^Median (range), **Clavien-Dindo classification ≥ grade 2

### Dietary Intake Changes

The median DI (range) of the overall population before surgery, at 1 month after surgery, and at 3 months after surgery, was 1713.5 kcal/day (1126–2330), 1541.5 kcal/day (986–2195), and 1638.5 kcal/day (816–2443), respectively. At 1 month postoperatively, the median % DI loss (range) of the overall population was −9.3% (−46.3% to 38.5%), and the median % NI (sugar, protein, and lipid) loss (range) was −12.4% (−85.2% to 99.6%), −4.0% (−39.3% to 108%), and −8.9% (−48.4% to 51.2%), respectively. At 3 months postoperatively, the median % DI loss (range) of the overall population was −3.6% (−50.8% to 54.1%), and the median % NI (sugar, protein, and lipid) loss (range) was −3.1% (−57.5% to 57.4%), −1.4% (−43.3% to 53%), and −1.5% (−49.6% to 83.0%), respectively.

**Fig. 1:**
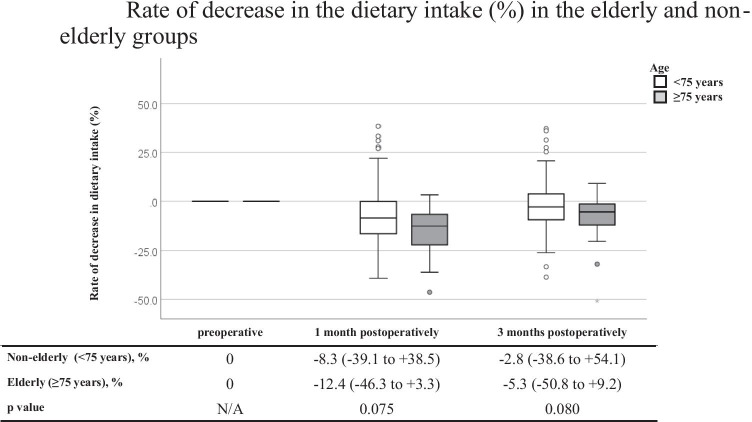
Rate of decrease in the dietary intake (%) in the elderly and non-elderly groups

**Fig. 2:**
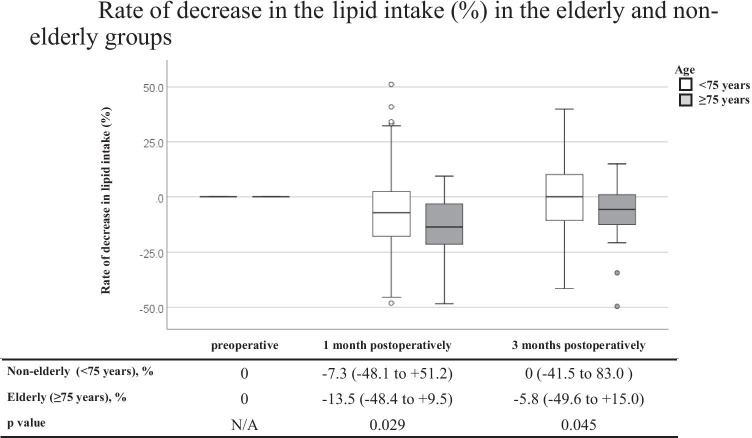
Rate of decrease in the lipid intake (%) in the elderly and non-elderly groups

**Fig. 3:**
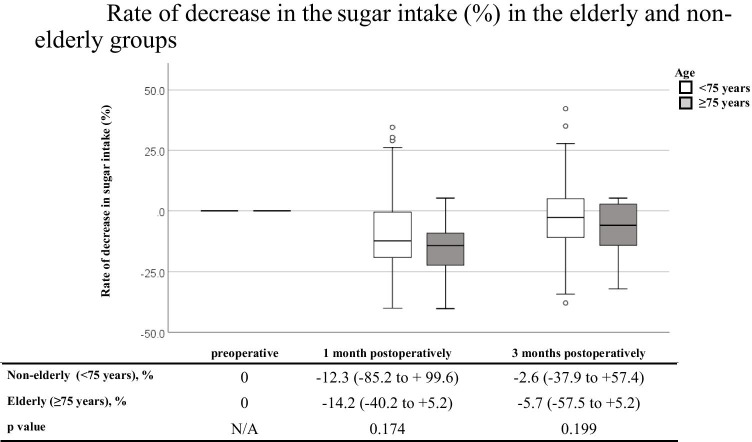
Rate of decrease in the sugar intake (%) in the elderly and non-elderly groups

**Fig. 4:**
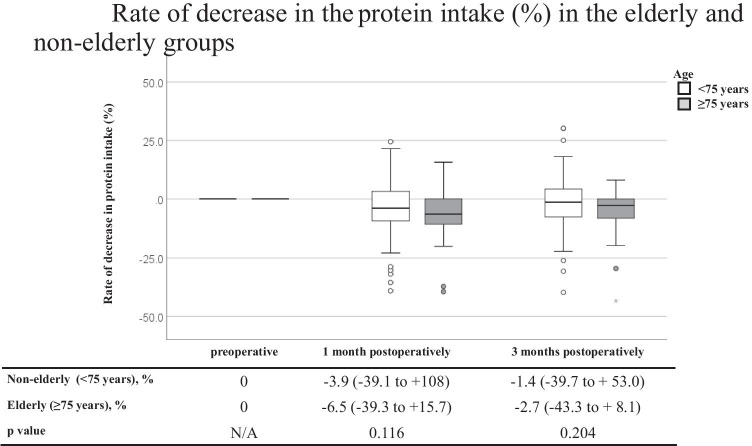
Rate of decrease in the protein intake (%) in the elderly and non-elderly groups

Table 3 shows a comparison of the DI and three major NI between the non-elderly and elderly groups. Before surgery, the DI and three major NI were similar between the two groups. Similarly, at 1 and 3 months postoperatively, there were no significant differences in the DI or three major NI between the two groups. Figure 1 shows a comparison of the % DI loss between the non-elderly and elderly groups at 1 and 3 months postoperatively. Marginally significant decreases were observed at 1 and 3 months postoperatively in the elderly group (*p* = 0.075 and 0.080, respectively). Figures 2, 3, and 4 show a comparison of the % three major NI loss between the non-elderly and elderly groups at 1 and 3 months postoperatively. Among them, the loss of lipid intake was significantly greater in the elderly group than in the non-elderly group at 1 and 3 months postoperatively (*p* = 0.029 and 0.045, respectively) (Fig. 2). On comparing the loss of sugar and protein intake after gastrectomy between the non-elderly and elderly groups, there were no significant differences between the two groups (Fig. 3, 4).

**Table 3:** Dietary intake and major nutrients intake

	All cases	Non-elderly group (< 75 years)	Elderly group (≥ 75 years)	*P* value
	Median, (range)	Median, (range)	Median, (range)	
	*N* = 150	*N* = 127	*N* = 23	
**Preoperative term**				
Dietary intake, kcal/day	1713.5 (1126–2330)	1710.0 (1126–2256)	1721.0 (1256–2330)	0.744
Sugar intake, g/day	219.5 (137–307)	219.0 (137–307)	221.0 (161–301)	0.917
Protein intake, g/day	69.0 (44–83)	69.0 (44–83)	71.0 (51–80)	0.584
Lipid intake, g/day	53.0 (37–71)	53.0 (37–69)	52.0 (40–71)	0.788
**1 month postoperatively**				
Dietary intake, kcal/day	1541.5 (986–2195)	1547.0 (986–2195)	1413.0 (1018–2007)	0.126
Sugar intake, g/day	192.5 (31.2–513)	195.0 (31.2–513)	184.0 (143–273)	0.182
Protein intake, g/day	67.0 (39–135.2)	67.3 (39–135.2)	64.0 (40–82.2)	0.243
Lipid intake, g/day	49.0 (28–79.3)	49.0 (28–79.3)	45.0 (33–60)	0.056
**3 months postoperatively**				
Dietary intake, kcal/day	1638.5 (816–2443)	1652.0 (917–2243)	1544.0 (816–2081)	0.238
Sugar intake, g/day	211.0 (91.1–304)	212.0 (117–304)	207.0 (91.1–266)	0.297
Protein intake, g/day	68.0 (36.8–99.4)	68.0 (37–99.4)	67.0 (36.8–99.4)	0.204
Lipid intake, g/day	52.0 (29–78.7)	52.6 (29–78.7)	47.6 (29.2–65.0)	0.057

## Discussion

The present study quantified the changes in the DI of elderly patients after gastrectomy for gastric cancer compared with that in non-elderly patients and compared the changes in the intake of three major nutrients (sugar, protein, and lipid) after gastrectomy between elderly and non-elderly patients. The major finding was that the % DI loss after gastrectomy at 1 and 3 months postoperatively did not differ to a statistically significant extent between the non-elderly and elderly groups; however, the patients in the elderly group tended to experience greater % DI loss at 1 and 3 months postoperatively than those in the non-elderly group. Furthermore, on investigating the intake of three major nutrients, the patients in the elderly group experienced significantly more serious lipid intake loss at 1 and 3 months postoperatively than those in the non-elderly group. Taken together, these findings suggest that serious lipid intake loss should be considered in order to suppress DI loss in elderly patients after gastrectomy.

In the present study, the loss of DI after gastrectomy tended to be greater in elderly patients at 1 and 3 months postoperatively than in non-elderly patients. There are several possible reasons for this result. The first possible reason involves the age-related loss of activities after surgery. Amemiya et al. examined the postoperative recovery of the physical condition, activities daily living (ADL), and quality of life (QOL) and identified predictors for the functional recovery of patients ≥ 75 years old who underwent elective surgery for gastric or colorectal cancer [21]. They indicated that age was a risk factor for a protracted decline in the ADL. Elderly patients experience a loss of activity after gastrectomy, and the energy requirements for the whole day may decrease because of this decreased activity. The second possible reason involves the age-related decrease in bowel function. Several reports have demonstrated the existence of aged-related changes in the gastrointestinal tract [22–25]. Esophageal motility may reduce the reduction of neurons in the mesenteric plexus in older people [22]. Gastric motility is impaired with aging [23], but the small intestine is unaffected [24]. With aging, colonic motility can be influenced by signal transduction pathways and cellular mechanisms that control smooth muscle contraction [25]. These age-related reductions in the bowel function may decrease the DI after gastrectomy in elderly patients. The third possible reason is prolonged surgical stress in elderly patients. Endocrine reactions to surgical stress have been reported to differ between elderly and non-elderly patients [26, 27]. When surgical stress occurs, immune cells produce cytokines that act as mediators of both immune and systemic responses to injury. Roubenoff et al. examined the production of cytokines and serum C-reactive protein in elderly patients [27]. They showed that the production of interleukin-6 and interleukin 1 receptor antagonist was increased in elderly patients and that dysregulation of some inflammatory cytokines occurred with age. Due to this dysregulation, the effects of surgical stress may be prolonged in elderly patients compared with younger ones. These factors may influence the more serious DI loss after gastrectomy in elderly patients than in non-elderly patients.

Furthermore, the present study indicated that elderly patients experienced significantly more serious lipid intake loss at 1 and 3 months postoperatively than did non-elderly patients. Regarding why this result was observed, the first possible reason is that the lipid intake of elderly patients may be naturally lower than that of non-elderly patients. Conventionally, lipid digestion and absorption is considered to decline with age [28]. However, recent studies suggest that lipid digestion and absorption are probably normal in healthy aging humans and animals [29]. A similar result was observed in the present study, wherein the lipid intake before surgery was similar between the elderly and non-elderly patients. Therefore, this first possible reason may not be valid. The second possible reason is that lipid digestion and absorption may decline more seriously after gastrectomy in elderly patients than in non-elderly patients. However, Holt et al. indicated that lipid absorption was probably normal in healthy aging humans and animals, and that aging alone may not impair lipid absorption to a clinically significant extent [29]. They also suggested that the presence of inter-current complicating illness or the stress of acute event, such as surgery, major dietary change, or travel, might result in intestinal malfunction. After gastrectomy, it is generally recognized that various clinical problems may occur, including various abdominal and systemic symptoms, restriction of food intake, weight loss, and a decrease in physical activity, which are collectively labeled as post-gastrectomy syndrome (PGS); these issues can negatively influence the quality of life (QOL) after gastrectomy [30–32]. Various factors that may underlie PGS have been considered, including the loss of the reservoir function, decreased secretion of gastric acid and gastrin and ghrelin, changes in the food passage route, vagotomy to dissect lymph nodes, and pancreatic and biliary exocrine insufficiency [31]. Furthermore, recent studies have demonstrated that advanced age is a factor associated with poorer symptom scale after gastrectomy [33]. The lipid digestion and absorption are similar between elderly and non-elderly patients before gastrectomy, but malfunction of digestion and absorption of lipids may occur after gastrectomy in elderly patients. As a result, elderly patients may experience more serious lipid intake loss after gastrectomy than non-elderly patients.

Several limitations associated with the present study warrant mention. First, the present study was conducted at a single hospital and had a small sample size. The results need to be confirmed in another cohort or in a prospective multicenter study with a larger sample size. Second, the DI was quantified using the FFQW82, which cannot directly measure the DI. However, it was difficult to measure the DI directly after gastrectomy at home; thus, we needed to measure the DI by an alternative method. The methods most suitable for calculating the DI after gastrectomy should be investigated. Third, we failed to assess the severity of symptoms, living status, and QOL of patients after gastrectomy. Elderly patients may experience a malfunction of digestion and lipid absorption after gastrectomy, which may result in the deterioration of the symptom severity, living status, and QOL. These factors might influence the DI after gastrectomy. Finally, in the present study, our institution is a specialized cancer center. Thus, there might be patient’s selection bias in the present study. The patients had good performance status. Patients with poor performance status (e.g., performance status ≥ 3, severe dementia, and dysphagia) could not be treated in our hospital, as we specialize in cancer treatment. Thus, there may have been selection bias.

In conclusion, the loss of DI after gastrectomy tended to be greater in elderly patients at 1 and 3 months postoperatively than in non-elderly patients. On investigating the loss of the intake of three major nutrients, elderly patients were found to have experienced significantly more serious lipid intake loss at 1 and 3 months postoperatively than non-elderly patients. Our results suggest that serious lipid intake loss should be considered in order to suppress DI loss in elderly patients after gastrectomy.
